# Nanostructured Materials for Solar Cell Applications

**DOI:** 10.3390/nano12010026

**Published:** 2021-12-23

**Authors:** Katsuaki Tanabe

**Affiliations:** Department of Chemical Engineering, Kyoto University, Nishikyo, Kyoto 615-8510, Japan; tanabe@cheme.kyoto-u.ac.jp

The use of nanomaterials in technologies for photovoltaic applications continues to represent an important area of research. There are numerous mechanisms by which the incorporation of nanomaterials can improve device performance. We invited authors to contribute original research articles or comprehensive review articles covering the most recent progress and new developments in the design and utilization of nanomaterials for highly efficient, novel devices relevant to solar cell applications. This Special Issue aimed to cover a broad range of subjects, from nanomaterials synthesis to the design and characterization of photovoltaic devices and technologies with nanomaterial integration.

Yanbin Wang, Changlong Zhuang, Yawen Fang, Hyung Do Kim, Huang Yu, Biaobing Wang and Hideo Ohkita of Changzhou University, China and Kyoto University, Japan presented “Improvement of Exciton Collection and Light-Harvesting Range in Ternary Blend Polymer Solar Cells Based on Two Non-Fullerene Acceptors” [1]. Alvien Ghifari, Dang Xuan Long, Seonhyoung Kim, Brian Ma and Jongin Hong of Chung-Ang University, Korea presented “Transparent Platinum Counter Electrode Prepared by Polyol Reduction for Bifacial, Dye-Sensitized Solar Cells” [2]. Tianyi Shen, Qiwen Tan, Zhenghong Dai, Nitin P. Padture and Domenico Pacifici of Brown University, USA presented “Arrays of Plasmonic Nanostructures for Absorption Enhancement in Perovskite Thin Films” [3]. Qiang Zhang, Shengwen Hou and Chaoyang Li of Kochi University of Technology, Japan presented “Titanium Dioxide-Coated Zinc Oxide Nanorods as an Efficient Photoelectrode in Dye-Sensitized Solar Cells” [4]. Yasushi Shoji, Ryo Tamaki and Yoshitaka Okada of National Institute of Advanced Industrial Science and Technology, Japan and The University of Tokyo, Japan presented “Temperature Dependence of Carrier Extraction Processes in GaSb/AlGaAs Quantum Nanostructure Intermediate-Band Solar Cells” [5]. Panus Sundarapura, Xiao-Mei Zhang, Ryoji Yogai, Kazuki Murakami, Alain Fave and Manabu Ihara of Tokyo Institute of Technology, Japan and Univ Lyon, France presented “Nanostructure of Porous Si and Anodic SiO_2_ Surface Passivation for Improved Efficiency Porous Si Solar Cells” [6]. Mao-Qugn Wei, Yu-Sheng Lai, Po-Hsien Tseng, Mei-Yi Li, Cheng-Ming Huang and Fu-Hsiang Ko of National Chiao Tung University, Taiwan and Taiwan Semiconductor Research Institute, Taiwan presented “Concept for Efficient Light Harvesting in Perovskite Materials via Solar Harvester with Multi-Functional Folded Electrode” [7]. Kodai Kishibe, Soichiro Hirata, Ryoichi Inoue, Tatsushi Yamashita and Katsuaki Tanabe of Kyoto University, Japan presented “Wavelength-Conversion-Material-Mediated Semiconductor Wafer Bonding for Smart Optoelectronic Interconnects” [8]. The guest editor would like to thank all of the authors of this Special Issue for their contributions to its successful completion.
